# Atypical Presentation of Salzmann Nodule: A Case Report and Literature Review

**DOI:** 10.7759/cureus.15397

**Published:** 2021-06-02

**Authors:** Mohanna Y Aljindan, Malak A Bamashmoos, Reem A AlShamlan, Amal A AlOdaini, Hanoof A Alabdullatif

**Affiliations:** 1 Ophthalmology, King Fahd University Hospital, Imam Abdulrahman Bin Faisal University, Dammam, SAU; 2 Ophthalmology, Dahran Eye Specialist Hospital, Dammam, SAU; 3 Ophthalmology, College of Medicine, Imam Abdulrahman Bin Faisal University, Dammam, SAU; 4 Histopathology, King Fahd University Hospital, Imam Abdulrahman Bin Faisal University, Dammam, SAU

**Keywords:** atypical salzmann, salzmann nodular degeneration, literature review, central salzmann, visually significant salzmann

## Abstract

Salzmann’s nodular degeneration (SND) is an unusual corneal condition that is slowly progressive and non-inflammatory in nature. It results in millimetric gray-white to bluish nodules formation anterior to Bowman’s layer of the cornea. It usually affects both eyes in 80% of the cases. These elevated nodules are located near the limbus or in the mid-peripheral cornea, with some exceptions. Salzmann nodule develops following corneal trauma or inflammation. However, it can present idiopathically.

Here, we report an atypical case of idiopathic symptomatic large central SND that was treated successfully with superficial keratectomy.

## Introduction

Salzmann’s nodular degeneration (SND) is an unusual corneal condition that is slowly progressive and non-inflammatory in nature. It results in millimetric gray-white to bluish nodules formation anterior to Bowman’s layer of the cornea [1]. It usually affects both eyes in 80% of the cases [2]. These elevated nodules are located near the limbus or in the mid-peripheral cornea with some exceptions. Salzmann nodule develops following corneal trauma or inflammation. However, it can present idiopathically [1,3-5].

In this report, we present a case of an atypical Salzmann nodule located in the center of the cornea covering the visual axis for which it was treated surgically.

## Case presentation

**Figure 1:**
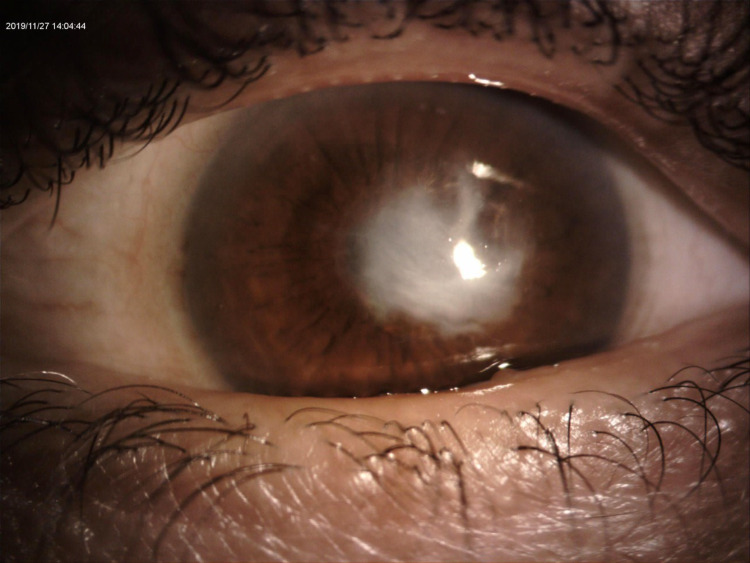
Slit lamp photo of the left eye showing large central elevated whitish lesion

**Figure 2:**
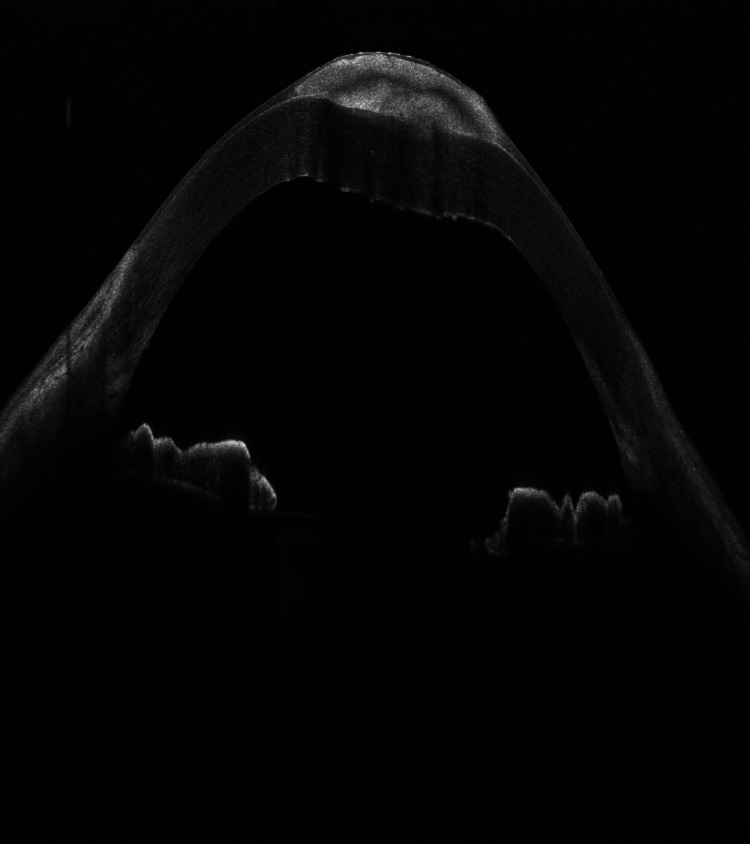
OCT showing hyper-reflective sub-epithelial lesion anterior to the Bowman's layer

**Figure 3:**
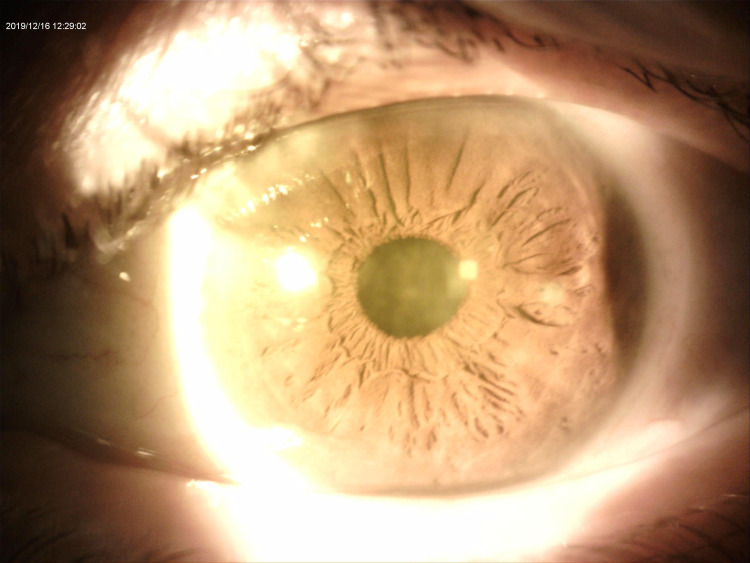
Slit lamp photo of the cornea after excision of the lesion with mild haze

**Figure 4:**
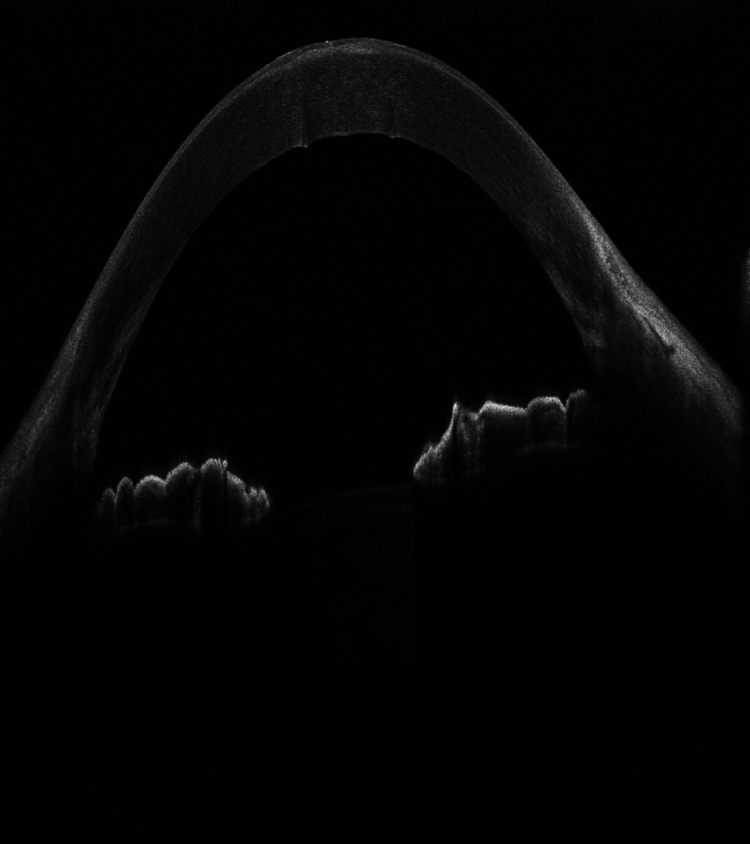
OCT of the cornea after excision of the lesion

**Figure 5:**
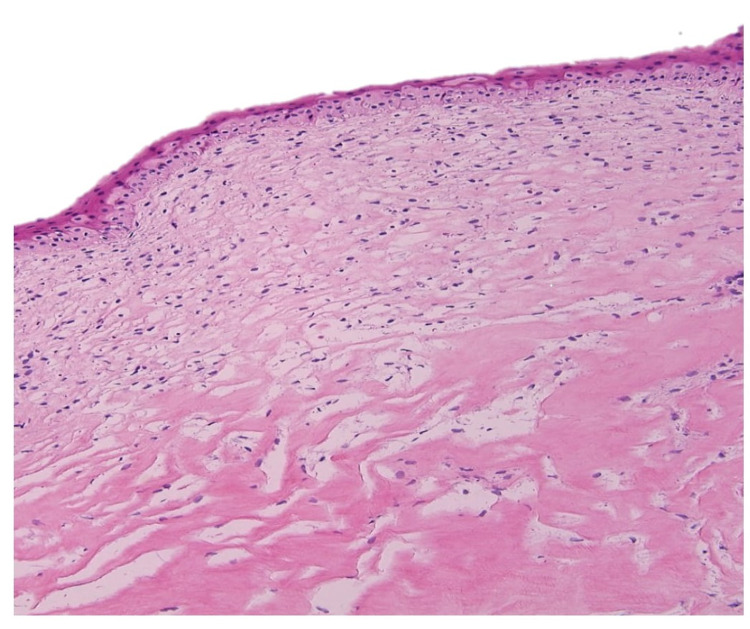
Histopathologic examination shows thinning of the corneal epithelial lining with sub-epithelial fibroblastic proliferation and deposition of hyalinized material (hematoxylin and eosin x100)

A 54-year-old Saudi male patient, who is unknown to have any medical illness presented to the ophthalmology clinic complaining of decreased vision in both eyes, more pronounced in the left eye for two years that was getting progressively worse in the last five months. He denied any previous ocular infection or trauma and never used contact lenses. The patient did not undergo any ocular surgery nor used any eye drops. His examination revealed visual acuity of 0.15 (in decimal) (20/200) in the right eye and hand motion in the left eye. The slit-lamp examination of the right eye showed multiple peripheral sup-epithelial round whitish lesions, and the left cornea showed a single central lesion (Figure 1). A cross-sectional photo of anterior segment optical coherence tomography (OCT) of the same eye showed a central hyper-reflective corneal lesion that is confined to the subepithelial layer (Figure 2). The clinical impression was left corneal keloid or Salzmann’s nodule. Superficial keratectomy with manual excision of the lesions was done for both eyes as means of treatment and to compare the samples (Figures 3, 4). The patient’s vision improved post-operatively; it reaches 0.2 (20/100) in the right eye and 0.3 (20/60) in the left eye. Histologically, the cornea demonstrated thinned epithelium overlaying the nodule with fibroblastic proliferation and deposition of extracellular hyalinized material. Neither an inflammatory infiltrate nor hemosiderin deposits were identified. Descemet membrane and endothelium were not present in the specimen. These findings, although non-specific, are consistent with the clinical diagnosis of Salzmann’s nodule (Figure 5).

## Discussion

SND usually presents as symmetrical bilateral nodules located more commonly in the periphery of the cornea [4,5]. Our patient had asymmetric nodules bilaterally with an atypical central, visually significant nodule in the left eye.

SND was first described in 1925 by Maximilian Salzmann as multiple or solitary bluish-white corneal nodular elevation. Then it was named in 1930. Multiple studies followed his series to describe and understand the disease more [6,7]. Although the pathogenesis of SND is still unknown, recent studies have shown that there is a major role in metabolically active basal cells of the nodular epithelium [8].

SND is typically associated with chronic corneal disorders, meibomian gland dysfunction, contact lens wear, surgical procedure, traumatic injury of the cornea, peripheral vascularization, pterygium, keratoconjunctivitis sicca, or inflammatory diseases like Crohn's disease. However, several cases of SND presented without any previous ocular diseases as seen in our patient [3,8,9]. SND usually affects females. A retrospective study conducted between 1996 and 2002 showed that 89.2% of patients with SND were female [9]. SND can be presented with decreased visual acuity, epiphora, photophobia, foreign body sensation, or asymptomatically in 15% of cases [4,8].

Histologically, the findings of SND include the presence of subepithelial fibrosis, fibroblastic degeneration, absent or disrupted bowman’s layer, deposition of extracellular matrix, and attenuated overlying corneal epithelium [6,10,11]. These findings were similar to what was found in our case. The histopathologic features are nonspecific and the diagnosis of (SND) should be made with clinical correlation [4,10-12].

Salzmann's nodule is managed conservatively or surgically depending on the patient's presentation. Conservative management includes treating known underlying causes, proper hygiene, warm compressor, lubricant, and steroid drops [4]. Most patients benefit from medical treatment alone. However, there are some indications for surgical interventions like; decreased visual acuity or foreign body sensation [13]. Superficial keratectomy alone or with alcohol, mitomycin-C, or amniotic membrane transplant were documented in the literature as surgical options. Lamellar keratoplasty is rarely needed [5]. Around 90% of patients will have an improvement in visual acuity after surgical intervention [14]. In our case, Salzmann's nodule was centrally located in the left eye and affecting the vision. Therefore, superficial keratectomy was done to clear the visual axis. The recurrence of SND is increasing with longer follow-up [9]. Most commonly, patients take five to 15 years to develop SND again. However, visually significant Salzmann nodule is uncommon as it affects 5% to 20% of those with the recurrence [9,14]. Table 1 summarizes cases documented in the literature.

**Table 1:** Cases documented in the literature

Reference	Case	Previous history	Location	Management
Garg, Sharma, & Khan (2019) [15]	A case report of a 50-year-old male	Medically free, presented with a progressive decrease in vision	Paracentral extending centrally, unilateral	Superficial keratectomy
Yang, Al-Hashimi, & Rootman (2018) [16]	A case report of 30- year- old female	Hyperthyroidism and post uncomplicated LASIK eight years back	Peripheral, bilateral Tearing, itching, ocular surface sensitivity, and dry eyes	Medical treatment d propylthiouracil (PTU), artificial tear use, loteprednol etabonate ophthalmic gel, eyelid taping, and selenium supplementation
Stem & Hood (2015) [17]	A case report of a 41-year-old female	Post uncomplicated LASIK for myopia	Peripheral unilateral with epithelial ingrowth centrally but not invade the visual axis	Superficial keratectomy
Lange, Bahar, Sansanayudh, Kaisermann, & Slomovic (2009) [18]	A case of a 50-year-old female	Progressive vision reduction for several years. Medical history of Crohn’s disease on infliximab with no eye involvement	Bilateral. Midperipheral	Visual field was not affected, follow up only
Sinha, Chhabra, Vajpayee, Kashyap, & Tandon (2006) [19]	Two case report: 1-Recurrent Salzmann's nodule of 40-year-old male following keratoplasty in both eyes, 22 years ago. 2-Recurrent Salzmann's nodule or 24-year-old male following keratoplasty in both eyes, six years ago.	Chronic trachoma and continued exposure to dust, wind and sunlight, probably were the predisposing factors.	Central bilateral	Treated with keratoplasty
Das, Link, & Seitz (2005) [1]	Case series of 30 eye	Five previous Keratitis, one ocular Trauma	40% peripheral 60% paracentral and central	Eight asymptomatic no surgical or medical intervention. 22 with abnormal visual acuity treated by PTK
Swann & Shuley (1989) [20]	A case report of a 32-year-old male	Vernal conjunctivitis	Unilateral peripheral	Asymptomatic did not require any treatment
Katz (1930) [7]	A case report of a 38-year-old female	Recurrent history of inflammation since the age of 14-year-old increases in severity at age of 32. (eczematous keratoconjunctivitis)	Unilateral peripheral	Mild mercurous chloride insufflation, mercuric oxycyanide (1:3,000), one drop three times a day, and yellow mercuric oxide ointment (3%), nightly

## Conclusions

This report highlights an unusual presentation of SND of a central idiopathic nodule affecting the vision. Superficial keratectomy for histopathologic examination can be very supportive in diagnosing atypical cases with large or central nodules.
